# Interoperation of an UHF RFID Reader and a TCP/IP Device via Wired and Wireless Links

**DOI:** 10.3390/s111110664

**Published:** 2011-11-08

**Authors:** Sang Hoon Lee, Ik Soo Jin

**Affiliations:** 1 Department of Electronic Engineering, Kyungnam University, 449 Woryeong-dong, Changwon, Korea; 2 Department of Information and Communication Engineering, Kyungnam University, 449 Woryeong-dong, Changwon, Korea; E-Mail: isjin@kyungnam.ac.kr

**Keywords:** UHF RFID, RFID reader, RFID tag, RF coordinator, ZigBee, interrogator, TCP/IP, UHF antenna

## Abstract

A main application in radio frequency identification (RFID) sensor networks is the function that processes real-time tag information after gathering the required data from multiple RFID tags. The component technologies that contain an RFID reader, called the interrogator, which has a tag chip, processors, coupling antenna, and a power management system have advanced significantly over the last decade. This paper presents a system implementation for interoperation between an UHF RFID reader and a TCP/IP device that is used as a gateway. The proposed system consists of an UHF RFID tag, an UHF RFID reader, an RF end-device, an RF coordinator, and a TCP/IP I/F. The UHF RFID reader, operating at 915 MHz, is compatible with EPC Class-0/Gen1, Class-1/Gen1 and 2, and ISO18000-6B. In particular, the UHF RFID reader can be combined with the RF end-device/coordinator for a ZigBee (IEEE 802.15.4) interface, which is a low-power wireless standard. The TCP/IP device communicates with the RFID reader via wired links. On the other hand, it is connected to the ZigBee end-device via wireless links. The web based test results show that the developed system can remotely recognize information of multiple tags through the interoperation between the RFID reader and the TCP/IP device.

## 1. Introduction

The radio frequency identification (RFID) technology was originally developed by the US military for the purpose of missile tracking. An RFID is a radio frequency system that has a tag (or a transponder) consisting of an electronic microchip and a reader (or interrogator) for reading and writing data. The data exchange between the tag and the reader is achieved by using magnetic or electromagnetic fields. The production data stored in the microchip can be transferred over a distance of more than 10 m by the antenna. After decoding the tag data, the reader sends the result to the host computer. Because the RFID has a relatively long transmission range and a multi-tag recognition ability, it has the potential to become a core replacement technology for the conventional barcode systems used in the fields of safety, security, and logistics [1–3].

RFID technology is a non-contact method for information identification using radio frequency. Continuous growth of the Internet, the manufacture of low-cost tags, and standardization of electrical identification codes has led to several practical industrial applications of this technology. RFID technology uses several frequency ranges, such as low frequency (LF, 30 ∼ 300 KHz), high frequency (HF, 3∼30 MHz, typically 13.56 MHz), ultra-high frequency (UHF, 0.3 ∼ 3 GHz), and microwave (>3 GHz) and is classified accordingly as LF RFID, HF RFID, UHF RFID, and microwave RFID, respectively. The advantage of using LF RFID systems is that these systems are cost effective, but they have short recognition ranges (<60 cm). On the other hand, the advantage of using HF RFID systems is their relatively big recognition range (∼60 cm), but these systems are expensive. Among the above mentioned classes of RFID systems, UHF RFID systems are potentially suitable for many applications despite of the high cost of the tags. The UHF band offers longer reading distances (3.5 m ∼ 10 m) than other RFID frequency bands because of a relatively compact high-gain antenna. A relatively high transmitter power is also permitted at an ultra-high frequency. Since modulated backscattering rather than inductive coupling is used for the signal processing, a tag antenna is simple and has a low manufacturing cost. The electromagnetic skin depth is also shorter at an ultra-high frequency than at lower operating frequencies. This enables the use of low-cost printed antennas and relatively thin metallization layers, which can save additional material costs [4,5]. Some useful information regarding the design of UHF RFID systems is available in the scientific literature. For instance, the best selection of key control, transmission, and inception parts is presented in [6]. Some design issues in UHF RFID IC and back-end circuits are discussed in [7,8]. The experimental performance of an UHF RFID system in real applications is discussed in [9,10]. Moreover, an FPGA-based system design for a UHF RFID reader is presented in [11].

This RFID technology already has many commercial applications, such as preventing automobile theft, gaining entrance to buildings, dispensing goods, and managing logistics. As RFID technology is deployed in transition from closed networks to standards-based open networks, interoperability with equipment made up of multiple devices is required. Users can minimize their total cost by setting up networks that provide this interoperability in a combined network that supports wired and wireless communication links. Rather than operating separately, RFID devices could be more effective by operating within an integrated network. As the volume of tag data that is created and transmitted, and the potential uses of the information will be growing, it can be expected that the role of RFID-related networks will become much more important. For example, although Cisco Systems is not building RFID tags or reader products, the company intends to work with RFID technology providers to help enable interoperable, end-to-end solutions [12].

In this study, we focus on a system co-implementation of a UHF RFID reader and a TCP/IP device via wired and/or wireless communication channels. This combined system can support functionality such as network availability and scalability. We have used a suitable Web-based test-bed and have performed the experiments using a set of prototypes. The objective of this study is to establish remote interoperation between the UHF RFID reader and the TCP/IP device via wired and/or wireless links. The wireless connection between the UHF RFID reader and the TCP/IP device is realized by using a ZigBee end-device. We also present in this paper the simulation results for a UHF antenna.

## 2. System Design

**Figure 1.**
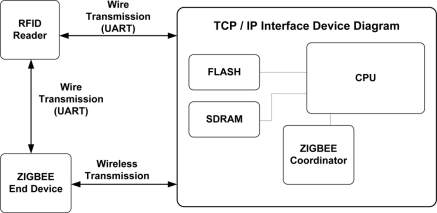
System configuration.

Figure 1 shows the system configuration for interoperation between a UHF RFID reader and a TCP/IP device. The proposed system consists of three functional blocks: an RFID reader, a ZigBee end-device, and a TCP/IP interface device. A UHF RFID reader can communicate with a TCP/IP device by two methods. One is through a direct connection with the TCP/IP device via UART, and the other is through an indirect connection with the TCP/IP device via a ZigBee end-device.

ZigBee protocols define a type of sensor network for mainly residential and commercial applications such as heating, air conditioning and light control. Whereas Bluetooth is geared towards user mobility and eliminating cabling between short-distanced devices, ZigBee is more oriented towards remote control and automation. ZigBee networks take advantage of low data rates (∼up to 250 kbit/s), low power consumption (∼longer battery lifetimes of up to 10 years), and low cost (∼$1.5 per module) compared to Bluetooth networks. Since the proposed system has been specially adapted for useful features such as low cost, low power, and remote control, we used a ZigBee device for the wireless transmitting link in our proposed system.

### 2.1. UHF RFID Reader

**Figure 2.**
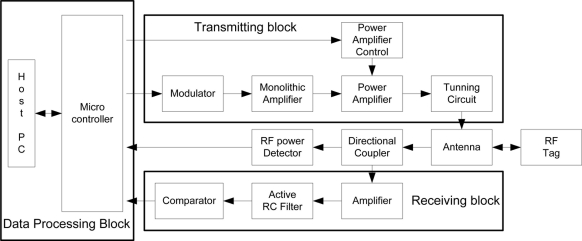
Block diagram of UHF RFID reader.

Figure 2 shows the functional block diagram of a 900 MHz RFID reader. The RFID reader consists of three functional blocks: a data processing block, a transmitting block, and a receiving block. The transmitting block sends request commands to an RF-tag in the recognition field. The receiving block receives data from RF-tags through an antenna. The data processing block deals with the tag information.

The transmitting block contains a signal generator, a modulator, a power amplifier, and a tuning circuit. The signal generator generates the carrier signals for the RFID system. In this study, we generate a 915 MHz signal from a 10 MHz crystal using an SI4113-BM frequency synthesizer. In order to design the modulator and the power amplifier, we use an RFMD RF2173 chip because this device has an output power of +36 dBm and a gain of 32 dB. Further, we control the output power level by using an EXAR MP7524A D/A converter via a microcontroller.

The receiving block consists of a detector, an amplifier, a filter, and a comparator, as shown in Figure 2. The detector splits the tag signal received from the antenna into two parts by using a directional coupler, an Alpha DC08-73 chip. The amplifier LT6200CS6 having a gain bandwidth of 165 MHz enhances the tag signal from the directional coupler. The amplified signal is supplied to the comparator, LMV7219M7, through an active RC filter, LT1568.

**Figure 3.**
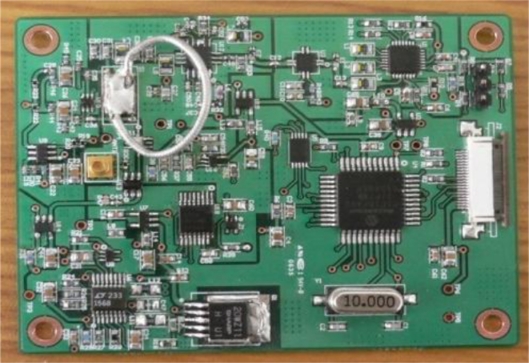
Prototype of UHF RFID reader.

The data processing block is operated by a microcontroller PIC18F452 with a built-in 10-bit A/D converter. Figure 3 shows the prototype of the UHF RFID reader, and Table 1 presents the design details.

### 2.2. ZigBee End-Device

**Figure 4.**
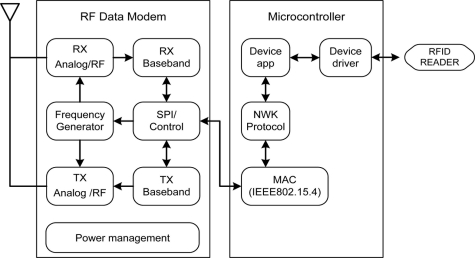
Block diagram of ZigBee End-Device.

**Figure 5.**
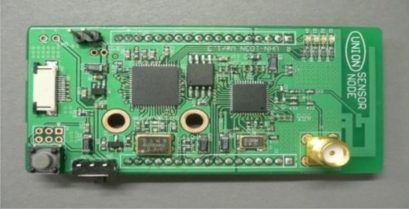
Prototype of ZigBee End-device.

Figure 4 shows the functional block diagram of a ZigBee end-device, which contains an RF data modem, a microcontroller, and a power management system. We used a CC2420 RF transceiver, manufactured by Chipcon, for ZigBee communication. The chip is a 2.4 GHz IEEE802.15.4 compliant RF transceiver designed for low-power communication. Further, we used an ATmega-128L RISC processor for the microcontroller’s operation. The data communication between the RF modem and the microcontroller is carried out through SPI. Figure 5 shows the fabricated ZigBee end-device.

### 2.3. TCP/IP Device

**Figure 6.**
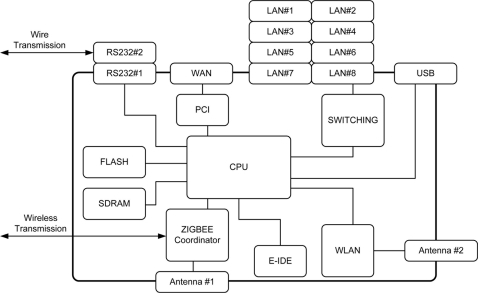
Block diagram of TCP/IP device.

**Figure 7.**
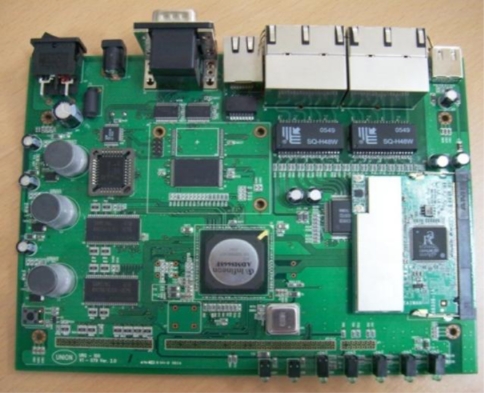
Prototype of TCP/IP device.

A TCP/IP device can be used as a gateway for the remote management of a UHF RFID reader. The TCP/IP device can be directly connected with the RFID reader via UART or indirectly via a ZigBee end-device as shown in Figure 1. The role of TCP/IP device is basically to provide the function that can remotely handle tag data between a RFID reader and a terminal located on far away. When the TCP/IP device and the RFID reader are connected together with a wired type connection, the TCP/IP device transmits the achieved tag data to a near or far terminal directly, but in the case of a wireless connection, the tag data from the reader can be achieved by wireless type communication by the ZigBee coordinator in the TCP/IP device. Since the ZigBee coordinator is compatible with IEEE802.15.4, it can communicate with another ZigBee end device on the RFID reader side, therefore remote management of the tag information under various circumstances can be carried out by the interoperation between the RFID reader and the TCP/IP device. In this paper, we designed a TCP/IP device based on the ADM8668 reference board manufactured by Infineon. Figure 6 shows the functional block diagram of the designed TCP/IP device. Figure 7 shows the fabricated TCP/IP device.

### 2.4. UHF RFID Reader Antenna

**Figure 8.**
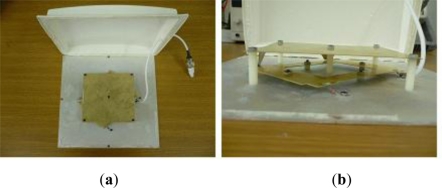
UHF RFID antenna: (**a**) top view and (**b**) side view.

Figure 8 shows the as-fabricated UHF antenna. The antenna has two layers: the top layer is made of copper, and the bottom layer of aluminum. The junction between the top layer and the bottom layer is connected to a 50 Ω film resistor. The antenna is enclosed by ABS plastic housing and is 13 × 13 cm^2^ in size. The antenna is designed to ensure a circular polarization with a center frequency of 915 MHz, an upper frequency of 950 MHz, and a lower frequency of 840 MHz. The circular polarization antenna can transmit and receive signals from more than two channels with one frequency. Further, the antenna offers good transparency to obstacles, is robust against multipath interference, and has a low polarization loss.

**Figure 9.**
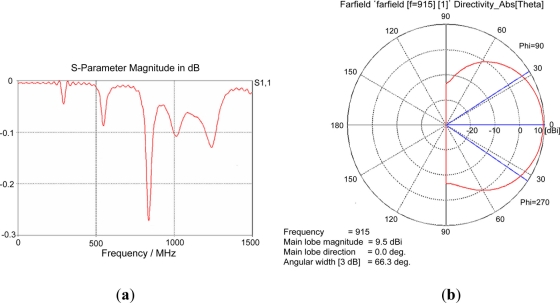
Simulation results of UHF antenna: (**a**) S_11_ parameter and (**b**) radiation pattern.

Figure 9 shows the simulation results of the fabricated UHF antenna. Figure 9(a) shows the simulation results of the S_11_ parameter. The narrowband reflection property between 800 MHz and 900 MHz is observed in the simulation. Figure 9(b) shows the results in the form of a radiation pattern. The maximum gain of the designed antenna is calculated as 9.5 dBi.

## 3. Experimental Results

**Figure 10.**
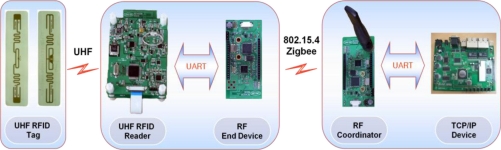
Series of prototypes.

Figure 10 shows a series of the prototypes fabricated using commercial parts. The image on the right shows the TCP/IP device and the RF coordinator. The image in the middle shows the UHF RFID reader and the ZigBee end-device, and the image on the left is of the antenna tag. The RFID reader and the TCP/IP device are connected with an IEEE802.15.4 ZigBee wireless channel. On the other hand, the reader and the RF end-device are linked with a wired UART. The TCP/IP device and the RF coordinator are also connected with a wired UART.

**Figure 11.**
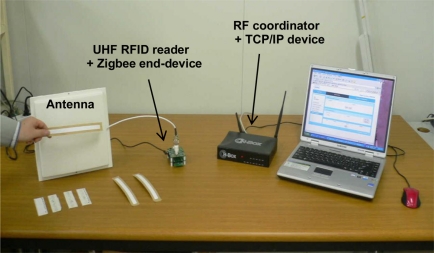
Test setup for interoperation between RFID reader and TCP/IP device.

Figure 11 shows the test setup for the recognition of the UHF tag and the management of the recognized tag information. The test is divided into three parts in order to verify the results of these apparatus: tag recognition by the UHF RFID reader, transmission of the tag information by the ZigBee end-device and the RF coordinator, and remote management by the TCP/IP device.

**Figure 12.**
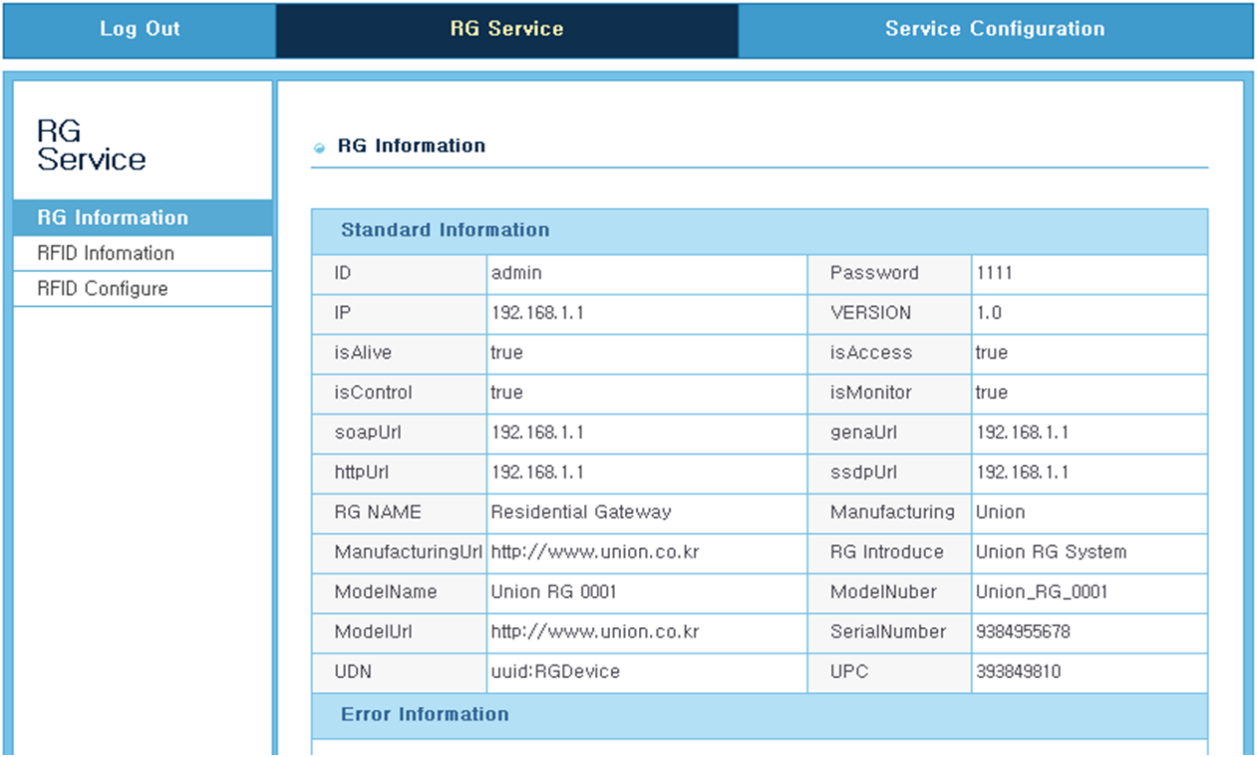
Web GUI window for recognition of the TCP/IP device.

**Figure 13.**
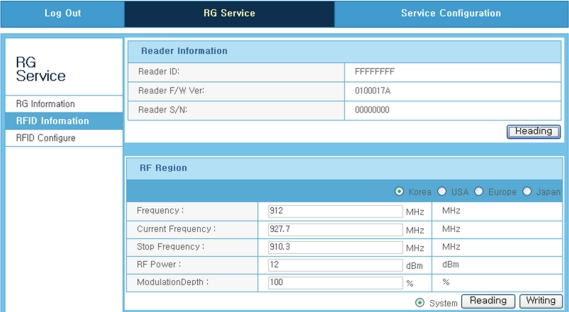
Web GUI window for recognition of the RFID reader.

**Figure 14.**
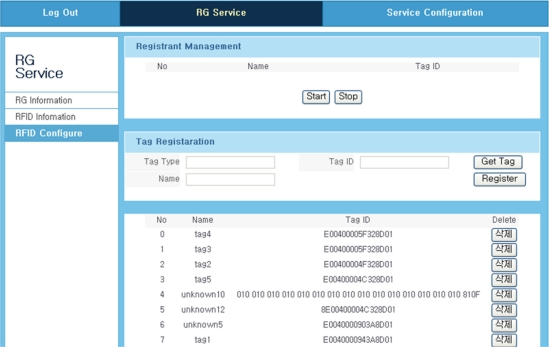
Tag recognition results.

Figure 12–14 shows the GUI window for the remote RFID recognition under a Web-based test circumstance. The procedure of the interoperation between the RFID reader and the TCP/IP device is carried out sequentially step by step. The first step is to confirm the information of the TCP/IP device which is connected with the service network. The next step is also to confirm the information of the RFID reader. The last process is to recognize information of tags registered from the RFID reader. Of course the RFID reader can newly register unregistered tags. Figure 12 shows the GUI screen for recognizing the TCP/IP device (RG; resident gateway). The information such as IP address, serial number and operation status, has shown in this window.

Figure 13 shows the GUI screen image for recognizing the RFID reader. This screen supports to user basic information such as reader ID, firmware version, and reader serial number. Moreover, user can also know technical information such as operating frequency, RF power, and modulation depth.

Figure 14 shows the recognized tag information on the GUI screen image. These tags can be registered or removed by using register and delete buttons.

## 4. Conclusions

In this paper, we introduced a system implementation of a UHF RFID reader and a TCP/IP device. The UHF RFID reader operating at 915 MHz was compatible with EPC Class-0/Gen1, Class-1/Gen1 and 2, and ISO18000-6B. The TCP/IP device was prepared to retransmit the tag data for remote management. The ZigBee end-device and the RF coordinator could support a wireless link between the RFID reader and the TCP/IP device. To verify the operation of the designed prototypes, we used a Web-based test case. The fabricated prototypes can be used in application fields such as remote inventory management and ubiquitous sensor networks (USNs).

## Figures and Tables

**Table 1.** Design summary of UHF RFID reader.

Power Capability	600 mA @ 500 mW/250 mA @ 15 mW/<50 μA @ Sleep
RF Power	12–27 dBm, 1-dBm step
Read Range	1 m @ int. antenna/2 m @ ext. antenna
Host Interface	TTL, 12C, SPI
